# Tongue Squamous Cell Carcinoma in a Young Patient Without Classical Risk Factors: A Case Report and Literature Review

**DOI:** 10.7759/cureus.110723

**Published:** 2026-06-12

**Authors:** Zainab Moudahik, Ziad Farih, Khaoula Bououdar, Mokrane Khazana, Youssef Naji

**Affiliations:** 1 Oral Surgery, Mohammed VI University of Sciences and Health, Casablanca, MAR; 2 Pathology, Université Hassan II, Casablanca, MAR

**Keywords:** case report, head and neck neoplasms, oral cavity squamous cell carcinoma, oral tongue cancer, risk factors, young adult male

## Abstract

Squamous cell carcinoma of the tongue usually affects older patients with established risk factors such as tobacco and alcohol use. Its occurrence in very young patients without classical risk factors remains clinically challenging and may lead to diagnostic delay. We report the case of a 20-year-old male patient with no history of tobacco or alcohol use who presented with a three-month history of lingual swelling, dysphagia, right-sided otalgia, marked weight loss, and deterioration of general condition. Clinical examination revealed a firm right submandibular lymphadenopathy and an ulcerative-exophytic, indurated lesion of the right lateral border of the tongue, associated with reduced tongue mobility. Magnetic resonance imaging showed a necrotic tongue mass crossing the midline, with right jugulocarotid lymph node involvement. Biopsy confirmed a moderately differentiated, keratinizing, infiltrative squamous cell carcinoma. Positron emission tomography (PET) imaging showed no distant metastatic disease. The tumor was considered locally advanced, and the patient received multimodal oncologic treatment. Despite an initial partial response, the patient died during follow-up. This case highlights the need for early biopsy and heightened diagnostic vigilance when faced with any persistent lingual lesion, even in young patients without tobacco or alcohol exposure.

## Introduction

Squamous cell carcinoma is a malignant tumor arising from epithelial cells and accounts for more than 90% of oral cavity cancers [1]. It most commonly affects men over the age of 50, with a marked male predominance. Classical risk factors include tobacco use, chronic alcohol consumption, and, to a lesser extent, infection with human papillomavirus (HPV), particularly in oropharyngeal locations [2,3]. However, several recent studies have reported an increasing incidence of tongue carcinoma in young adults, including patients with no exposure to traditional carcinogenic factors. This trend suggests the possible involvement of novel etiological factors, whether genetic or environmental, which remain insufficiently characterized [4]. The tongue represents the most frequently affected site within the oral cavity. Tumors arising in this location are generally locally aggressive and exhibit a high potential for regional lymph node metastasis, thereby compromising both functional and vital prognosis [5]. Although the increasing occurrence of oral tongue squamous cell carcinoma in young adults has already been reported, the present case is noteworthy because it occurred in a 20-year-old non-smoking and non-drinking patient and was associated with aggressive local extension, early cervical lymph node involvement, and an unfavorable clinical course [4]. In young patients without classical risk factors, non-classical mechanisms such as genetic susceptibility, viral factors, chronic local irritation, and other molecular or environmental factors may be considered, although their role remains incompletely established [2,3,6]. In this context, the presence of a family history of colorectal cancer in the patient’s father may raise the question of an underlying cancer susceptibility, although no direct hereditary link with lingual squamous cell carcinoma can be established. We report this case to highlight the need for early biopsy and heightened diagnostic vigilance when faced with any persistent lingual lesion, even in young patients without an obvious carcinogenic background.

## Case presentation

Patient information

A 20-year-old male patient, a university student with no significant medical or surgical history and no history of tobacco or alcohol use, presented with a lingual swelling associated with dysphagia and right-sided otalgia, evolving over a three-month period. His father was being followed for colorectal cancer. The clinical presentation occurred in a context of general health deterioration, with marked weight loss (from 74 to 58 kg) and loss of appetite.

Clinical findings

**Figure 1:**
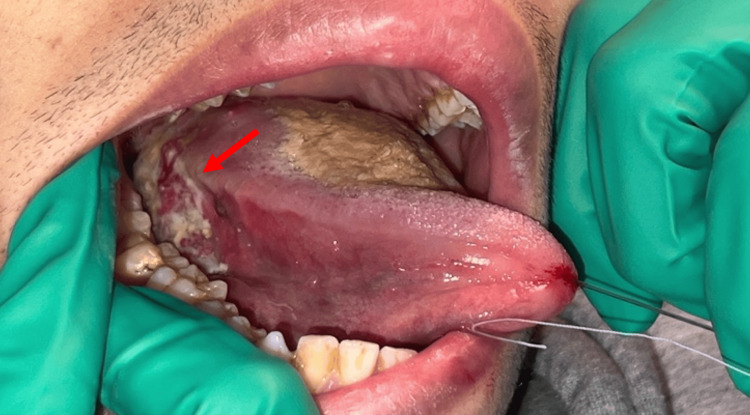
Intraoral photograph showing an ulcerative-exophytic lesion involving the lateral border of the tongue.

Extraoral examination revealed preserved facial symmetry with no abnormal temporomandibular joint sounds. A firm, slightly mobile right submandibular lymphadenopathy was noted. Intraoral examination revealed an infiltrative ulcerative-exophytic lesion on the right lateral border of the tongue, extending toward the tongue base, measuring approximately 3.5 cm in greatest diameter, with irregular and raised margins. Palpation demonstrated extensive induration extending toward the ipsilateral floor of the mouth, along with reduced tongue mobility, reflecting significant muscular invasion (Figure 1).

Diagnostic assessment

**Figure 2:**
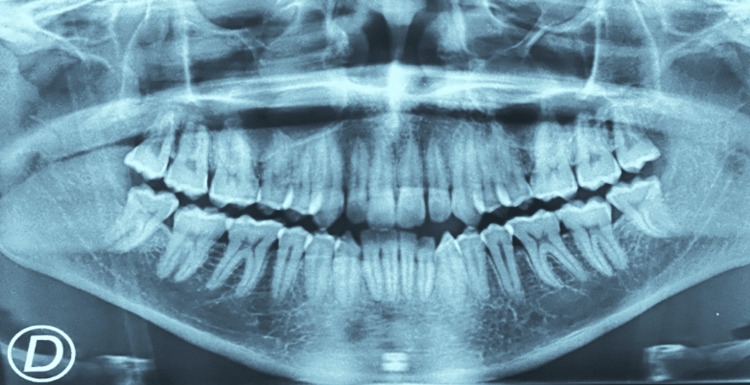
Panoramic radiograph showing preservation of mandibular bone structures.

At this stage, a diagnosis of squamous cell carcinoma was suspected. Complementary investigations were subsequently performed to assess the extent of the tumor. Panoramic radiography did not reveal any specific bone abnormalities but was considered an essential initial imaging assessment (Figure 2).

**Figure 3:**
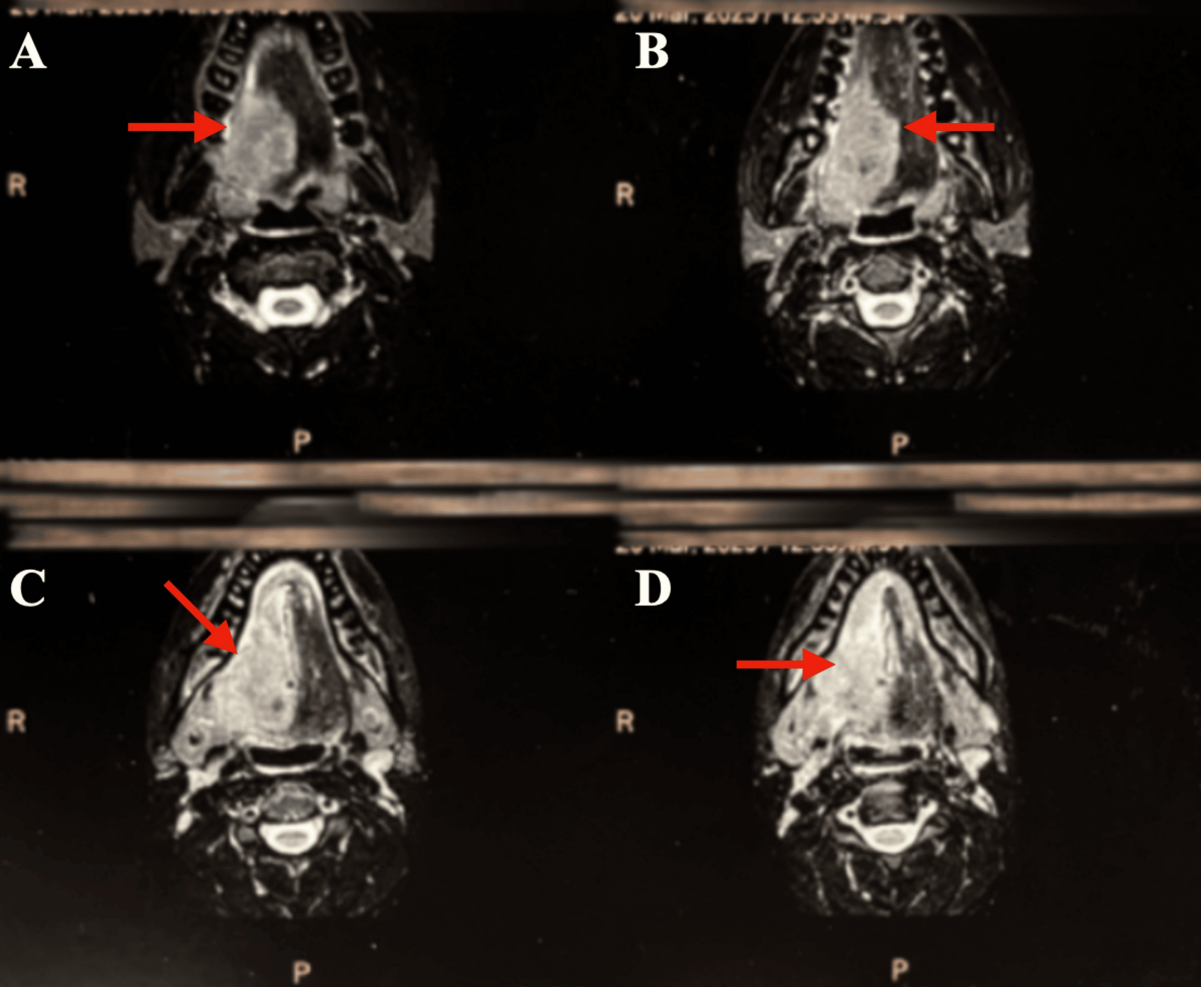
Axial MRI slices showing an infiltrative tongue mass. (A) Superior section demonstrating the upper extent of the lesion; (B) adjacent section showing that the lesion crosses the midline; (C) and (D) lower axial sections showing the lesion at its largest anteroposterior extension. MRI: magnetic resonance imaging.

Further evaluation was completed with magnetic resonance imaging (MRI), which demonstrated a necrotic soft-tissue tumor mass crossing the midline and extending posteriorly, associated with a right jugulocarotid lymphadenopathy measuring 12 mm (Figure 3).

**Figure 4:**
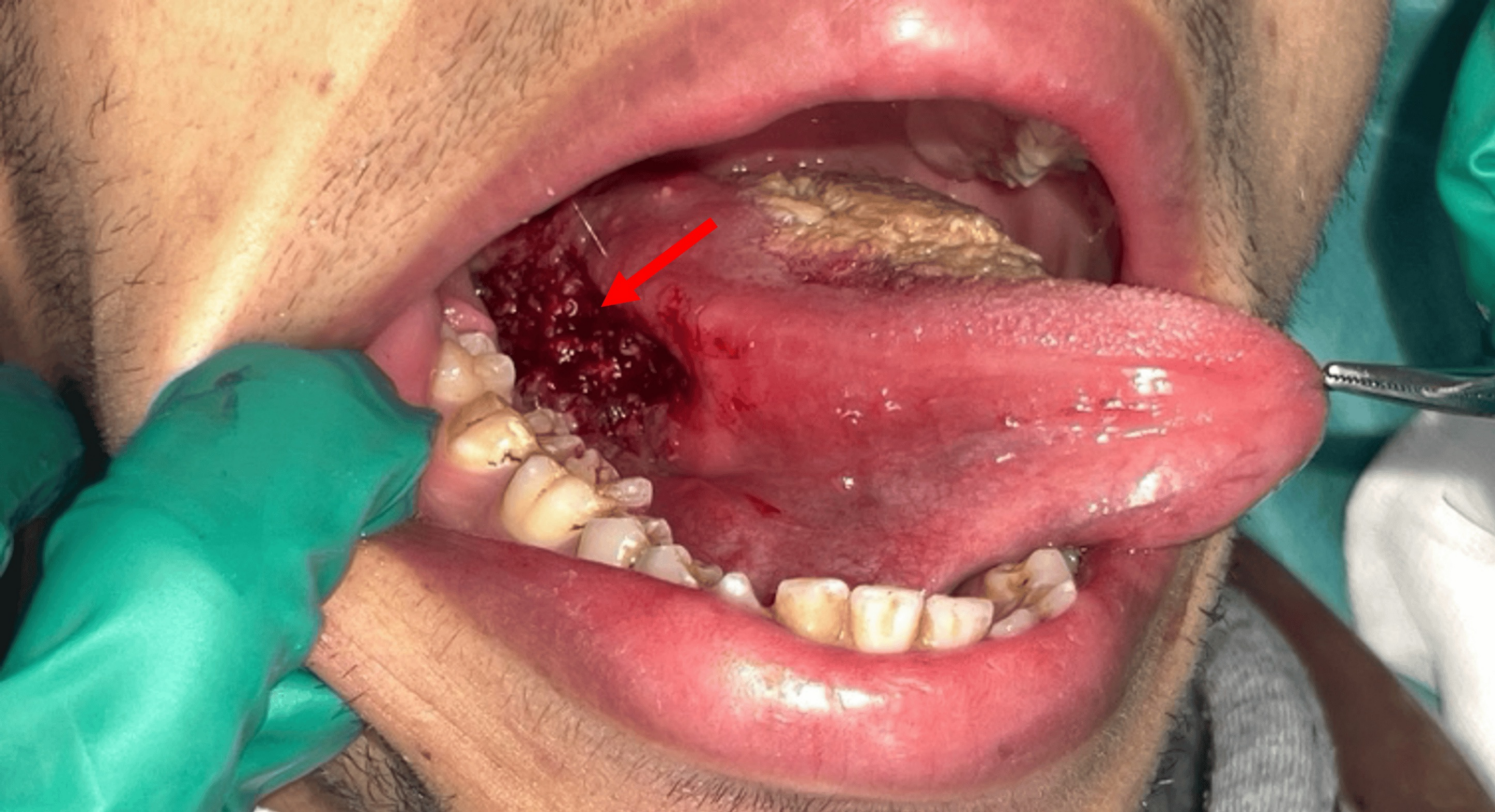
Post-biopsy intraoral photograph demonstrating persistent tumor infiltration and posterior extension.

**Figure 5:**
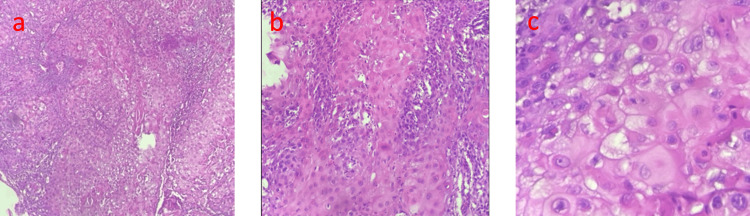
Moderately differentiated invasive squamous cell carcinoma with moderate pleomorphism (hematoxylin and eosin staining; original magnification ×100, ×200, and ×400). The tumor proliferation consisted of sheets and clusters of malignant polygonal cells with abundant eosinophilic cytoplasm, occasionally vacuolated, and exhibiting moderate cytonuclear atypia. The nuclei were enlarged, irregular, and hyperchromatic, with visible mitotic figures. Squamous differentiation was evidenced by the presence of intracellular keratinization foci and intercellular bridges, without identifiable keratin pearls, supporting the diagnosis of a moderately differentiated squamous cell carcinoma.

A biopsy was performed (Figure 4), and histopathological examination confirmed the presence of a moderately differentiated, keratinizing, infiltrative squamous cell carcinoma (Figures 5a-5c).

Positron emission tomography (PET) revealed intense hypermetabolic uptake in the lingual lesion and ipsilateral lymph nodes, with no evidence of distant metastatic disease. Laboratory investigations were otherwise unremarkable. According to the medical report, the tumor was clinically staged as T4N1M0 and histologically graded as a moderately differentiated, keratinizing, infiltrative squamous cell carcinoma. The disease was therefore considered locally advanced.

Follow-up and outcomes

**Figure 6:**
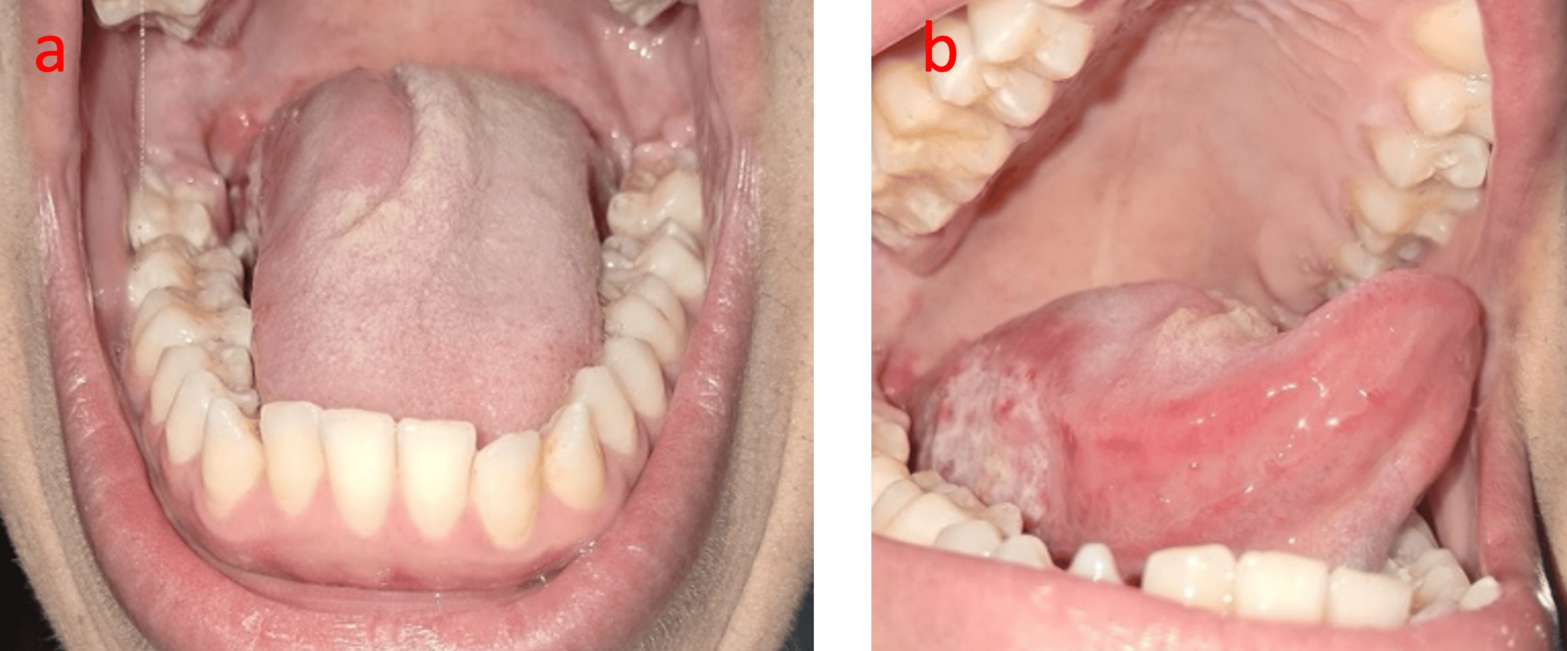
Clinical appearance after two cycles of chemotherapy, showing tumor regression.

Management was guided by tumor extent, disease stage, and the need to achieve optimal oncologic control while preserving orofacial functions as much as possible. Surgery remains the treatment of choice when complete resection with clear margins can be achieved. In the present case, the tumor was considered technically unresectable due to deep extension toward the floor of the mouth and involvement of the intrinsic and extrinsic tongue muscles. A combined therapeutic strategy consisting of chemotherapy and radiotherapy was therefore selected to ensure tumor control while limiting functional impairment and preserving the patient’s quality of life [7]. As part of pre-therapeutic dental preparation, comprehensive oral care was performed, including oral hygiene motivation, scaling with air polishing, conservative treatment of carious lesions, fabrication of fluoride trays, and regular follow-up. These measures are essential to reduce the risk of radiation-related complications, particularly mucositis, post-radiation caries, and osteoradionecrosis, in accordance with current recommendations. After two cycles of chemotherapy, a partial clinical response was observed, with a reduction in tumor size and improvement in pain symptoms. Clinical examination showed a decrease in the extent of the lingual ulceration without the appearance of new lesions (Figures 6a, 6b). The patient was treated with concomitant chemoradiotherapy, including external beam radiotherapy combined with weekly cisplatin (40 mg/m²) administered for six cycles as a radiosensitizing protocol. However, he experienced a deterioration in general condition, mainly related to treatment-associated adverse effects. Despite multidisciplinary management, the patient ultimately died seven months after the initial diagnosis.

## Discussion

Oral squamous cell carcinoma classically affects adults over the age of 45. However, cases occurring in young adults have been increasingly reported, although they still represent a minority. This population has drawn particular attention because of its atypical clinical profile and the frequent diagnostic delay, sometimes leading to diagnosis at an advanced stage [1]. The tongue is the most frequently involved site among intraoral squamous cell carcinomas. In young patients, these tumors appear to exhibit a more infiltrative behavior, with earlier muscular and cervical lymph node involvement, suggesting a potentially more aggressive biological profile. This observation justifies heightened vigilance during the clinical examination of any persistent lingual lesion in this population [4]. Tobacco and alcohol consumption represent the main risk factors for squamous cell carcinoma in middle-aged and older patients. However, the absence of these exposures in our patient underscores the atypical nature of this case and highlights that these cancers may occur outside the context of traditional risk factors. This finding is consistent with recent data reporting a growing proportion of young, non-smoking and non-drinking patients affected by squamous cell carcinoma. The association of young age and a family history of colorectal cancer in our patient raises the hypothesis of a genetic predisposition. Several studies have reported the involvement of germline variants affecting genes implicated in DNA repair mechanisms and cell cycle regulation, including CDKN2A, ATR, RECQL4, SDHB, as well as genes of the Fanconi anemia group (FANCA, FANCG). These alterations may account for the early onset of lingual squamous cell carcinoma in the absence of prolonged exposure to classical risk factors [8,9]. The prognosis of tongue squamous cell carcinoma remains generally guarded and depends on several well-established prognostic factors. Among the most important are tumor stage at diagnosis, depth of invasion, histological grade, cervical lymph node status, and surgical margin status. The presence of cervical lymph node metastases represents one of the most unfavorable prognostic factors, being associated with a significant reduction in overall survival and an increased risk of locoregional recurrence. Similarly, greater depth of invasion and higher histological grade are correlated with more aggressive tumor behavior [10]. In locally advanced forms, positive surgical margins and the presence of distant metastases are also associated with a poor prognosis. In young patients, some studies suggest a potentially more aggressive biological behavior, with higher rates of local recurrence in certain series, although the available data remain heterogeneous. A retrospective analysis from the National Cancer Database involving 3,262 patients under the age of 45 with tongue squamous cell carcinoma identified high histological grade, regional metastases, and increased depth of invasion as factors associated with reduced survival. In advanced disease, positive surgical margins and the presence of distant metastases were likewise correlated with decreased survival [11].

## Conclusions

Squamous cell carcinoma of the tongue in young adults is a rare but increasingly reported entity, often characterized by an atypical clinical profile and the absence of traditional risk factors. This case highlights the importance of maintaining a high index of suspicion for any persistent lingual lesion, regardless of patient age, and emphasizes the need for early biopsy to ensure timely diagnosis. Management of these tumors requires a multidisciplinary approach tailored to tumor stage and individual patient characteristics in order to achieve optimal oncologic control while preserving orofacial function. Furthermore, this case underscores the need for further research into the biological and genetic mechanisms involved in the early onset of tongue squamous cell carcinoma, with the aim of improving prevention, early detection, and therapeutic strategies.
